# Cryptosporidiosis-an overview

**DOI:** 10.1016/S1674-8301(11)60001-8

**Published:** 2011-01

**Authors:** Gordon J Leitch, Qing He

**Affiliations:** a Department of Physiology, and; b Department Microbiology, Biochemistry and Immunology, Morehouse School of Medicine, Atlanta, GA 30310-1495, USA

**Keywords:** *Cryptosporidium parvnm*, *Cryptosporidium hominis*, life cycle, *Cryptosporidium* infections, pathophysiology, immunological response, diagnosis, treatment, cryptosporidiosis, China

## Abstract

Apicomplexan protozoan parasites of the genus *Cryptosporidium* infect the gastrointestinal tract and lungs of a wide variety of animals, including humans. The majority of human infections are due to either *Cryptosporidium* *hominis (C. hominis)* and/or *Cryptosporidium parvum (C. parvum)*. The parasite has a complex life cycle that includes both asexual and sexual stages. While there are invasive free living stages, proliferation and differentiation take place within a unique parasitrophorous vacuole under the host cell brush border but outside the host cell cytoplasm. Infection is spread by environmentally resistant spores that primarily contaminate drinking water and occasionally food sources, which may cause significant outbreaks of diarrhea that generally lasts less than 2 w in immunocompetent individuals. In immunodeficient or immunosuppressed individuals, diarrhea may be copious and can result in significant morbidity and mortality, particularly in AIDS patients. Although diagnosis is relatively simple, effective drug treatment, particulary for infections in immunodeficient patients, has not been uniformly successful. This overview summarizes the species known to infect humans, aspects of the parasite life cycle, sources of infection, the pathophysiology of cryptosporidiosis, the immune response to infection, diagnosis, treatment and some aspects of cryptosporidiosis in China.

## INTRODUCTION

*Cryptosporidium muris (C. muris)* was first described by Tyzzer in 1910 and *Cryptosporidium parvum (C. parvum)* was described two years later[1]. However, it was not until the 1970s that *Cryptosporidium* was determined to be a significant cause of gastrointestinal disease in humans[2]. The genus *Cryptosporidium* is a member of the phylum *Apicomplexa*, which includes in its rank of parasitic protists other significant human pathogens such as *Plasmodium, Toxoplasma, Isospora, Sarcocystis, Cyclospora* and *Babesia*. Like the other members of this phylum, *Cryptosporidium* has a complex life cycle with both asexual and sexual stages and invasive stages that have the characteristic apical complex from which the phylum name is derived.

*Cryptosporidium* species has been found to infect mammals, birds, reptiles, amphibians and fish. The two species that most commonly infect humans are *Cryptosporidium* *hominis (C.* *hominis)* and *C. parvum*, and while the former species seems to be primarily limited to humans, the latter has a wide range of hosts, including most major domestic livestock animal species[3]. In humans, cryptosporidiosis mainly involves infection of the jejunum and ileum, resulting in a watery diarrhea lasting up to 2 weeks with the potential for recurrence. In immunodeficient and immunosuppressed individuals the infection may not resolve and may involve the biliary tree, stomach and lungs. The persistent diarrhea and malabsorption can become life-threatening, particularly in acquired immunodeficieney syndrome (AIDS) patients.

## *CRYPTOSPORIDIUM* SPECIES AND THEIR HOSTS

Because of the morphological similarities between the *Cryptosporidium* species, the wide host range of some species and the polymorphism within species, morphological, biological and molecular data are required to assign a *Cryptosporidium* isolate to a given species. To date, isolates have been assigned to 19 species using such data, and many more have been assigned to a genotype or subtype based on molecular data[3].

**Table 1:** *Cryptosporidium* species reported to infect humans

Species	Hosts	References
*C. parvum*	Mammals, humans	3,4,5,6,7,9,10
*C. hominis*	Predominantly humans	3,4,5,6,9,10
*C. muris*	Rodents, farm animals, humans*	3,4,5,7
*C. andersoni*	Livestock, humans*	3,5,10
*C. suis*	Livestock, humans*	3,6
*C. wrairi*	Guinea pigs, humans*	3,7
*C. felis*	Cats, humans*	5,6,8
*C. canis*	Dogs, humans*	3,6
*C. meleagridis*	Birds, humans	3,6,9,10

*Isolated human cases.

In spite of the advances that have been made in the study of cryptosporidiosis, there is not full agreement on the taxonomy of the various *Cryptosporidium* species. The worldwide distribution of this parasite, and the large numbers of genotypes, subtypes and subtype families already described[3]–[5],[11],[12] contribute to the challenge of developing a clear understanding of the molecular epidemiology of human cryptosporidiosis. Nevertheless, it appears that *C. hominis* (Type I) is primarily limited to humans and its transmission is therefore usually anthroponotic, while transmission of *C. parvum* (Type II) found in many mammals, particularly livestock, is usually zoonotic.

## *CRYPTOSPORIDIUM* SPECIES LIFE CYCLE

The major features of the life cycle of *C. parvum* or *C. hominis* are that it begins with the ingestion of fully sporulated, environmentally resistant oocysts. After excystation in the upper small intestine, the released sporozoites penetrate the mucus layer and attach to nearby enterocytes, causing them to form a parasitophorous vacuole around the parasite, which then differentiates into a trophozoite. An unusual feature of this vacuole is that it is located within the host cell plasma membrane, but outside the host cell cytoplasm, separated from the latter by a so-called feeder organelle and a specialized concentration of host cell cytoskeletal elements. Mitotic division of the parasite at this point results in a type I meront and the production of 6 or 8 merozoites. The merozoites resemble sporozoites. They escape the parasitophorous vacuole and attach to nearby enterocytes, establishing amplified asexual infectious cycles. Alternatively, the merozoite infection may result in a type II meront, and the production of 4 merozoites. As with the merozoites originating from type I meront, type II merozoites escape to infect nearby enterocytes, producing either a macrogamont (female) or a microgamont (male). Sixteen or more microgametes from the microgamont are released and each can fertilize a macrogamont to form a diploid zygote, which differentiates to an oocyst. Meiosis then results in 4 sporozoites being formed. This constitutes the sexual cycle, the end product of which is either a fully sporulated thin-walled oocyst (-20%) that excysts within the host and results in autoinfection, or a thick-walled oocyst (-80%) that is excreted into the environment.

### The oocyst

This fully sporulated thick-walled form (-5 µm in diameter) is resistant to prolonged environmental exposure in various water sources, and is also resistant to many commonly used disinfecting agents[13], including dilute bleach, which can be used when isolating viable oocysts from stool samples. *Cryptosporidium* oocyst wall proteins (COWPs) play a role in the environmental resistance of this and other apicomplexans[14]. Oocyst surface receptors also play a role in ensuring that this parasite stage is close to the host target tissue in the small intestine[15].

### Excystation

*In vitro*, bile salts and a temperature of 37°C are particularly effective at inducing excystation, while pancreatic enzymes are not[16]. So-called gastric species, *Cryptosporidium muris (C. muris)* and *Cryptosporidium andersoni (C. andersoni)*, are stimulated to excyst *in vitro* in an acidic environment or in the presence of taurocholic acid, while species that excyst in the upper small intestine, *C. hominis* and *C. parvum*, only excyst with the latter stimulus[17]. However, taurocholic acid is particularly effective if the oocysts are pre-acidified. Sporozoite-derived enzymes also play key roles in the excystation process.

### The sporozoite

Sporozoites are spindle-shaped (-4×0.6 µm). As with other apicomplexan parasites, the sporozoite apical complex plays essential roles in the gliding motility used by the parasite to access the target cell, target cell attachment and the establishment of the parasitophorous vacuole[18]. Sporozoite enzymes aid passage through the mucus blanket. *C. parvum* sporozoites move by a gliding motion powered by a parasite actin-myosin motor, leaving a trail[19] made up of components secreted by the apical complex micronemes, which also contribute to host cell selection[15]. Molecular and proteomic studies have identified several sporozoite proteins with potential roles in motility, and in host cell adhesion and invasion[15],[18],[20],[21].

### Adhesion to the host cell and establishment of the parasitophorous vacuole

The forward propulsion of the sporozoite results in its attaching to the apical surface of an enterocyte. There are several apical complex parasite protein candidates (of microneme, rhoptry and dense granule origin) that may play a role in the attachment[15],[18]. A sporozoite membrane-associated protein, CP47, is one such protein that binds to receptors on the target cell such as the p57 glycoprotein located on the ileal brush border[22]. Once attachment has occurred there is a general movement of the micronemes and dense granules with the extension of the rhoptry towards the attachment site. Many ultrastructural studies have shown that the attached sporozoite is engulfed by the host cell and adjacent microvilli elongate[23],[24]. This engulfing of the parasite and establishment of a parasitophorous vacuole involves swelling of the host cell as aquaporin I and the sodium-glucose symporter SGLTI are recruited to the host cell-parasite interface[24]. There is also considerable cytoskeleton remodeling in the area involving host cell actin polymerization. The signaling pathways involved in the invasion process and the establishment of the parasitophorous vacuole have been extensively studied[15],[18]. While it was previously believed that the parasitophorous vacuole was of host cell origin, it is now clear that it has significant parasite contributions[18]. In addition to an electron dense band at the host cell cytoplasm-parasitophorous vacuole interface, the parasite plasmalemma invaginates immediately above the terminal web, compresses, and becomes a highly folded membraneous structure often referred to as the feeder organelle[23] This structure is assumed to form the major pathway for parasite access to nutrients in the host cell cytoplasm, an assumption that is supported by the presence of ABC-cassette binding proteins[25]. Such a direct pathway to the host cell nutrients is very necessary for this parasite. The genomes of both *C. parvum* and *C. hominis* exhibit compaction and both species appear to have limited biosynthetic capabilities[26],[27] and must therefore rely on the host cell for nutrients.

### The trophozoite (1.5-2.5 µm in diameter)

After enclosure of the sporozoite within the parasitophorous vacuole, parasite dedifferentiation begins to occur, followed by differentiation[15]. As with the attaching sporozoite there is considerable derangement and elongation of the host cell microvilli immediately adjacent to the trophozoite.

### Type I meront

Mitosis within the trophozoites initiates the formation of type I meront within which 8 or 6 merozoites bud off from the residual body located near the parasite-host cell junction and feeder organelle. As with the trophozoite, the microvilli adjacent to type I meront are deranged and elongated. Merozoites are produced by budding from the schizont residual body and elongating. The parasitophorous vacuole membrane ruptures and type I merozoites escape[23].

### Type I merozoite

This stage is rod-like (0.4 ×1.0 µm), with a pointed apical region[28]. The motility, attachment to adjacent enterocyte apical membrane and formation of a trophozoite (i.e. establishment of the asexual cycle) are generally considered to be the same as or very similar to that of sporozoites. For example, CpSUB1, a sutilisin-like serine protein thought to play a role in invasion, is found at the apical pole of both sporozoites and merozoites[29].

### Type II meront

While type I merozoites go on to produce more type I meronts in a series of asexual cycles, some type I merozoites produce type II meronts. This stage ranges in size from 3-5 µm. The principal difference between this and type I meront is that only 4 merozoites develop in this stage. As with all *Cyptosporidium* intracellular stages, there is a well developed feeder organelle.

### Type II merozoite

Merozoites released from type II meronts are less uniform in shape, slightly larger and less active than those released from type I meronts.

### Microgamont and macrogamont

While some type II merozoites enter an enterocyte and produce a macrogamont, a spherical to oval structure of 4 to 6 µm in diameter with a large central nucleus, others produce a microgamont[30]. Nuclei bud from the microgamont residual body to form 16 separate rod-like non-flagellated microgametes (1.4×0.5 µm). These flagellum-free stages exit the microgamont and fertilize an adjacent macrogamont, resulting in the only diploid stage in the life cycle, the zygote.

### The zygote

The zygote develops into an oocyst, then undergoes sporogamy, all while still attached to the enterocyte apical membrane. As the oocyst differentiates, it becomes either a thin walled oocyst, or a thick walled oocyst. Those developing into a thick walled oocyst contain type I and type II wall-forming bodies[23]. Once they have differentiated, the oocyst detaches into the lumen to either re-infect the host in the case of thin walled-oocysts, or be excreted into the environment in the feces in the case of thick-walled oocysts.

It is possible but unlikely that, in addition to the life cycle described above, free extracellular stages may exist. While they have been described in *in vitro* cell culture models[28], there is no description of such extracellular stages in an *in vivo* model.

**Fig. 1:**
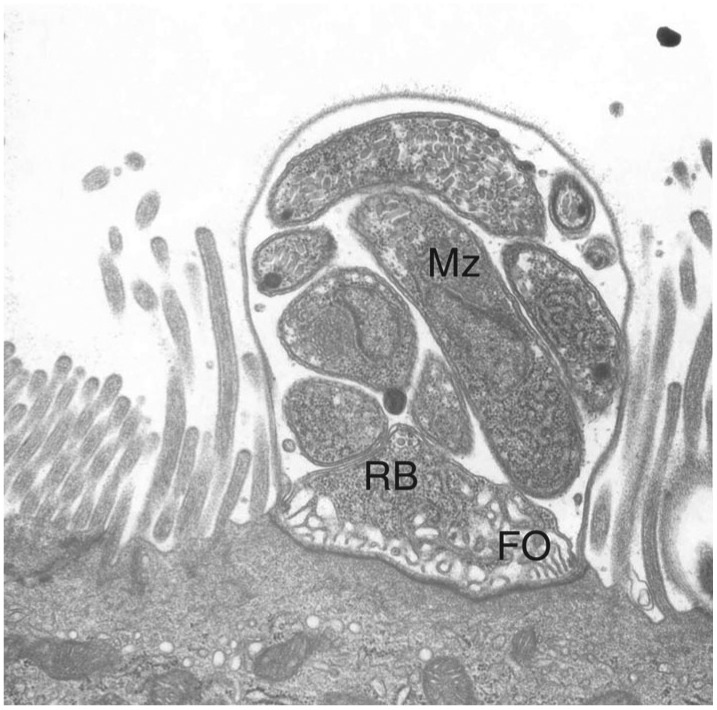
*Cryptosporidium pervum* type I meront in the jejunal epithelium of a nude mouse showing such characteristic features as an electron-dense desmosome-like boundary between host cell and parasitophorous vacuole, a pronounced feeder organelle (FO), residual body (RB), and merozoites (Mz). The enterocyte brush border microvilli immediately adjacent to the parasite are typically elongated.

***Fig. 1*** illustrates many of the features referred to above. A type I meront is seen containing merozoites. The feeder organelle lies above the electron dense boundary between host cell and parasitophorous vacuole, while the microvilli immediately adjacent to the parasite are elongated.

## *CRYPTOSPORIDIUM* SPECIES INFECTION

*Cryptosporidium* infections may be zoonotic or anthroponotic. Most of the major outbreaks of cryptosporidiosis have been attributed to contaminated drinking water[31], but oocysts have been recovered from food, such as fresh vegetables[32] and seafood[33], and person-to-person transmission may also be possible. The largest cryptosporidiosis outbreak reported to date in the USA occurred in Milwaukee, Wisconsin in 1993 when over 403,000 individuals were sickened out of a potentially exposed population of 1.6 million[31]. Longterm, the number of deaths resulting from cryptosporidiosis approximated 54, mostly AIDS patients. The outbreak was initially thought to be due to a failure in the drinking water purification system that occurred after runoff containing feces from infected cattle entered the system. However, subsequent genotyping of Milwaukee wastewater indicated that the predominant species was *C. hominis* (allele 1b), suggesting that the 1993 outbreak was anthroponotic rather than zoonotic[34]. Cost estimates of the economic impact of the Milwaukee outbreak (US$96 million in 1993 dollars for treatment and lost productivity costs) demonstrate the financial importance of guarding against such failures in water treatment[35].

Outbreaks of cryptosporidiosis have also been linked to contaminated recreational water, such as parks and swimming areas. In most human cryptosporidiosis studies, *C. hominis* is the major causative species. Individuals, particularly children, in rural areas may have a higher prevalence rate of *C. parvu*m infections than children in urban areas where *C. hominis* predominates[9],[36] and there is a greater *Cryptosporidium* species diversity in rural areas, in keeping with greater exposure to livestock and other animals[37]. In the United States, while many outbreaks may be anthroponotic *C. hominis* in origin, zoonotic *C. parvum* infections may be responsible for many of the reported sporadic cases. There are also geographical differences in the infections resulting from the different *Cryptosporidium* species. For example, in South America, there appears to be a higher incidence of *Cryptosporidium meleagridis* infections than elsewhere[6].

While a significant percentage of cases of *Cryptosporidium* infection may be asymptomatic, cryptosporidiosis is most frequently associated with diarrhea, nausea, vomiting, fever and abdominal discomfort that usually resolve within two weeks[38]. Children and the elderly are most affected[39],[40], and the younger the child is, the more severe the diarrhea is, with the age of peak prevalence being slightly different in different studies, but usually being less than 2 years of age. Malnutrition appears to increase the frequency of diarrhea and prolong the infection, even in immunocompetent children[37],[39],[40]. Early childhood infection has been shown to result in delayed growth, which may persist well beyond the period of infection[41],[42]. *C. hominis* appears to cause more severe acute disease[43], and more extraintestinal disease[6], and have more recurrent manifestations after an infection than do other *Cryptosporidium* species[44]. In addition to the more than 40 *Cryptosporidium* genotypes that have been described, *C. hominis* and *C. parvum* isolates have been assigned to subtypes and subtype families based on the molecular characterization of the 60 kDa glycoprotein (GP60) gene[45], and it appears that the clinical manifestations vary not only among the different *Cryptosporidium* species, but also among the *C. hominis* subtypes[6],[46]. In addition, *C. hominis* reinfection may occur more readily with a different subtype[46]. In a controlled study using human volunteers, oral challenge with oocysts of three different isolates of *C. parvum* had significantly different ID_50_s[47].

The incidence of cryptosporidiosis appears to depend not only on the species and subtypes of the *Cryptosporidium* oocysts, but on the environmental exposure, determined by such factors as season, availability of potable water and hygiene practices. In many, but not all studies the incidence peaks in the rainy season. The role of safe drinking water has been discussed above, and poor hygiene practices are well known to increase the risk of infection with all enteric pathogens.

As one would expect, there are great geographical and socioeconomic differences in the prevalence of cryptosporidiosis. In one seroepidemiological study, Zu *et al.*[48] compared serum anti-*Cryptosporidium* IgG levels in children less than 10 years of age in three villages in Anhui Province, China, with children less than 3 years of age in Forteleza, Brazil and children and young adults in Virginia, USA. They found an infection rate averaging 50% by the age of 10 years in the Chinese children, almost 100% by the age of 2 years in the Brazilian children, and 16.9% by young adulthood in the USA children.

In *Cryptosporidium*-infected immunodeficient individuals, such as AIDS patients and those with malignancies[49] and immunosuppressed transplant patients[50], diarrhea may become copious and unrelenting, resulting in wasting, while the water and electrolyte disturbances may be life-threatening, with up to 17 L/d of stool being reported[51]. Stool volumes in such individuals may therefore come close to those seen with cholera. Symptomatic bile and pancreatic duct and lung infections are more likely to occur in immunodeficient patients[52]. In general, a CD4^+^ T-cell count of <200/mm^3^ puts the patient at risk for a prolonged infection, and counts of <100/mm^3^ may result in an even more severe life-threatening diarrhea[53],[54]. However, as is the case with many cryptosporidiosis studies, there are substantial differences in clinical manifestations between infected individuals, and even patients with very low CD4^+^ counts may only exhibit transient diarrhea[55]. There is no doubt, however, that a *Cryptosporidium* infection increases the mortality rates in AIDS patients when comparing infected and uninfected AIDS patients with the same low CD4^+^count[54]. In an early UK study[55] *Cryptosporidium* positive HIV/AIDS patients were divided into those with transient infection (28.7%), chronic infection (59.7%), or fulminant infection or those patients with daily stool volumes >2 L (7.8%), and asymptomatic patients (3.9%). Those patients in the fulminant category all had CD4^+^ counts <50/mm^3^ and had the shortest survival time.

In the absence of any universally effective therapy for cryptosporidiosis, HIV/AIDS patients have shown marked improvement in their cryptosporidium-associated symptoms when antiretroviral therapy has been used to raise their CD4^+^ count[56]. The symptomatic improvement in patients seems to be more a function of the improved CD4 T lymphocyte count rather than a decrease in viral load, and intestinal mucosal CD4 cell numbers increase before both a rise in circulating CD4 lymphocytes and a decrease in *Cryptosporidium* infection. However, there is some question as to whether the infection is completely resolved in all such cases and whether a latent infection may cause a patient to develop severe diarrhea if the antiretroviral therapy is not continued for an extended period of time[56].

## PATHOPHYSIOLOGY

Much of the understanding of the pathophysiology of cryptosporidiosis has been the result of studies using calf, neonatal pig, primate and various neonatal or immunodeficient rodent models, and *in vitro* intestinal epithelial cell culture models. While cryptosporidiosis is most commonly a disease of the small intestine in immunocompetant individuals, extraintestinal gastric, hepatobiliary, pancreatic and pulmonary infections can cause signs, symptoms and even death in immunodeficient or immunosuppressed individuals. In one study of a group of *Cryptosporidium*-infected AIDS patients, 88.9% had parasite stages in the gastric mucosa[57]. The gastric mucosal abnormalities seen in *Cryptosporidium*-infected AIDS patients are highly variable and complicated by coinfections with other agents, making a correlation between symptomatology and infection difficult[58]. Antral infection and the resulting inflammation may be responsible for a partial reduction in gastric emptying. *C. muris* infects the stomach of a variety of animal models, resulting in gastric crypt dilatation, epithelial metaplasia and inflammation[59], but how such studies relate to immunocompetant and immunodeficient infected humans is unknown.

AIDS patients, transplantation patients, and individuals with primary immunodeficiencies [e.g. boys with X-linked immunodeficiency with hyper-IgM (XHIM)] are susceptible to biliary tract *Cryptosporidium* infections[60],[61]. HIV-1 Tat protein has been shown to decrease TLR4 expression in *in vitro* cholangiocytes, which may suppress the innate immune response, making the bile duct more susceptible to infection.[62] *Cryptosporidium* infection of the biliary tract and liver has been reported to cause a variety of hepatobiliary effects, including triaditis, and cholangitis, lobular hepatitis with periductal sclerosis, biliary sclerosis and necrosis with dilation in a variety of immunodeficient mouse models[63]. HIV replication and *Cryptosporidium* infection synergistically promote cholangiocyte apoptosis, and this apoptosis may account for some of the bile duct pathology. The low incidence of jaundice in these patients suggests that the bile duct is not completely occluded[63]. Nevertheless, it is not surprising that with many infected immunodeficient patients exhibiting cholangitis and papillary stenosis, there is a significant incidence of pancreatic involvement[64]. *Cryptosporidium* infection is known to cause both acute and chronic pancreatitis in immuncompetent and immunodeficient humans and animal models[64]–[66].

Pulmonary cryptosporidiosis has long been known to be a problem with birds[67]. It is also sometimes seen as a complication of intestinal cryptosporidioisis in humans, most commonly in immunodeficient patients where it can result in respiratory failure and death[68],[69]. In a recent Ugandan study of 9-36 month old human immunodeficienty virus (HIV)-seronegative children presenting with diarrhea, 35.4% of the children who had stool samples positive for *Cryptosporidium* also had positive sputum samples. Over half of these cases were not malnourished, arguing against malnutrition-induced immunodeficiency being a major contributing factor. This study suggests that the incidence of pulmonary cryptosporidiosis in immunocompetent children is higher than previously thought and the authors raise the possibility of a respiratory route for cryptosporidiosis transmission[70]. None of these studies indicate the pathophysiological mechanism underlying pulmonary cryptosporidiosis, but animal model studies clearly showed that the major target of the parasite is the tracheobronchial epithelium which showed malpighian metaplasia, with minimal parenchymal involvement[71]. The inflammatory response results in mucus hypersecretion and epithelial damage, perhaps mediated by an NF-κB-COX-2 mechanism[72].

Gastrointestinal cryptosporidiosis may affect the course of disease treatment by reducing the absorption of therapeutic agents. In one study of blood antiretroviral levels in AIDS patients, it was found that malabsorption caused by infection with either *Cryptosporidium* and/or *Isospora* reduced antiviral drug levels to subtherapeutic levels[73].

Three major mechanisms have been proposed for the diarrhea seen with cryptosporidiosis: 1) malabsorption resulting in an osmotic diarrhea; 2) parasite-induced generation of inflammatory products and host neurohumoral secretogogues; 3) secretory diarrhea resulting from a parasite enterotoxin. Different regions of the gastrointestinal tract have different absorptive and secretory profiles. While the small intestine, particularly the ileum, is the main site of a *Cryptosporidium* infection in immunocompetent individuals, the gastrointestinal parasite distribution is more complex and widespread in AIDS patients[74]. However, there is malabsorption in some patients, as evidenced by reduced vitamin B12 and D-xylose absorption in AIDS patients, and the malabsorption together with villus atrophy has been found to increase with increased parasite burden[75]. In one study of children of less than 2 years of age with cryptosporidiosis, there was evidence of mild enteritis, but the low fecal osmotic gap indicated that the diarrhea was secretory rather than osmotic in nature[76]. Similarly, in a group of *Cryptosporidium*-infected children with full-blown AIDS, the small osmotic gap also supported the concept that the diarrhea was secretory rather than osmotic in origin[77]. The mechanisms of intestinal secretory diarrhea have been summarized in two reviews[78],[79].

In general, the histopathology of *Cryptosporidium*-infected small intestine does not correlate well with the parasite load or clinical illness, particularly in humans[80]. In HIV-*Cryptosporidium* co-infected individuals, there is a decrease in both Paneth cell number and Paneth cell degranulation, suggesting a reduction in antimicrobial defensins in this population. As with other aspects of studies involving AIDS patients, it is difficult to determine which effects are due to a specific opportunistic infection and which are due to a combination of effects seen in severely immunodeficient individuals with multiple co-infections. In the case of the Paneth cell study, the degranualtion correlated with low body mass index and zinc deficiency, and thus might have been secondary to malnutrition[81].

In addition to human studies, experimental studies using neonates, immunodeficient and human xenograft animal models, and *in vitro* cell culture studies have yielded results which point to some common features in an intestinal *Cryptosporidium* infection. The most consistent observations include villus atrophy, crypt hyperplasia, infiltration of the lamina propria, chloride secretion, glucose malabsorption and a reduced barrier function (increased paracellular permeability)[80]–[84].

The loss of absorptive surface area is frequently seen in intestinal cryptosporidiosis[80]. Using an HCT-8 cell culture model, Mele *et al.*[85] found that in early *C. parvum* infections host cell apoptosis was inhibited as parasite development was initiated, while at later stages of the infection the apoptosis increased. Other studies have shown that infected enterocytes also exhibit necrotic changes[80]. Such phenomena are probably not limited to *C. parvum* and *C. hominis* as *C. andersoni* causes apoptosis and disruption of the zonula occludens (tight junction) in both human and bovine epithelial cell lines[86].

The various *in vivo* and *in vitro* models studies suggest that several of those factors involved in the production of secretory diarrhea[79] play a role in the diarrhea seen with cryptosporidiosis. For example, in a study using macaque jejunum, Hernandez *et al.*[87] demonstrated that substance P (SP), expression of the substance P receptor (NK1), glucose malabsorption and chloride ion secretion were all increased in *C. parvum* infected tissue. Use of a SP receptor antagonist reversed the physiological effects. Similar results were obtained using an immunosuppressed mouse model[88]. While SP is a neuropeptide found in the enteric nervous system, it is also produced by many inflammatory cells. Prostaglandins are known to directly and indirectly act through cholinergic and VIPergic neurons and *C. parvum* infection significantly increases prostaglandin production in piglet and human enterocyte models[89],[90]. However, in a long-term infection model using anti-interferon (IFN)-γ treated SCID mice, the intestinal transport effects of the experimental cryptosporidiosis were indomethacin resistant[91], suggesting that the observed effects were not prostaglandin dependent.

The evidence for a parasite-derived enterotoxin is minimal[80]. On the other hand, cytokines and chemokines elaborated by inflammatory cells in the lamina propria and by the mucosal epithelial cells of *Cryptosporidium*-infected individuals may contribute to the intestinal pathophysiology. Both tumor necrosis factor (TNF)-α and IFN-γ increase mucosal permeability[92], and TNF-γ increases enterocyte intestinal anion transport via a prostaglandin-mediated mechanism[93]. In a study using human volunteers infected with *C. parvum*, the expression of interleukin (IL)-1β and TNF-α in jejunal biopsies correlated with infection, but not with symptoms[94], while substance P levels did correlate with the severity of intestinal disease[95]. Inflammatory cells in the lamina propria appear to play a complex role in the pathophysiology of cryptosporidiosis. For example, neutrophils protect the mucosal barrier in piglet cryptosporidiosis[96]. The differences between animal models and the human infection and between cryptosporidiosis in healthy and malnourished or immunocompromised individuals have been discussed by Pantenburg and colleagues[97].

## IMMUNE RESPONSES TO *CRYPTOSPORIDIUM* SPECIES INFECTION

Both innate and adaptive immunity play critical roles in protecting against *Cryptosporidium* infection and in parasite clearance. Innate immune responses involve intestinal epithelial cells (IECs), IFN-γ and natural killer (NK) cells, nitric oxide (NO), toll-like receptor (TLR) pathways, antimicrobial peptides, prostaglandins, mannose-binding lectin, cytokines, chemokines, dendritic cells (DCs) and macrophages. The adaptive immune system is composed of highly specialized, cells, such as T cells and B cells and processes that eliminate or prevent pathogenic challenges.

### Innate immune system

#### IECs

IECs are the first cells invaded in cryptosporidiosis. IECs express pattern-recognition receptors, including TLRs and intracellular Nod-like receptors (NLRs) that recognize the parasites. IECs also express major histocompatibility complex (MHC) class I and class II molecules that are required for antigen processing and presentation[98]–[100]. It has been reported that CXCL10 is highly upregulated in the IECs of AIDS patients with active cryptosporidiosis[101]. IECs are also a cellular source of IL-18 (IFN-γ-induced factor). The *IL*-*18* gene is upregulated in the small intestine of mice in response to infection, and IL-18 mRNA and protein are upregulated in IECs infected with *C. parvum in vitro*[102],[103].

#### IFN-γ and nitric oxide synthase (iNOS)

IFN-γ is a significant player in the innate immune response against *C. parvum* as shown in nude mice and SCID mice. Although SCID mice are deficient in T- and B-lymphocytes, their resistance to *C. parvum* infection is still IFN-γ-dependent, which suggests that IECs may be another important source of this cytokine. In various cryptosporidiosis models the infected epithelium showed an increased production of NO by iNOS[104]–[108]. Deleting or inhibiting iNOS significantly exacerbated epithelial infection and oocyst shedding, while the administration of antioxidants has been shown to exacerbate *C. parvum* infection. IFN-γ can stimulate macrophages to produce iNOS-generated NO and activate stress signaling cascades including the c-jun-N-terminal kinase (JNK) pathway. These events activate an apoptotic cascade that ultimately results in cell death. In a piglet model iNOS expression promotes epithelial defense against infection by *C. parvum* in a NF-κB-dependent manner[108].

#### DCs

Limited work has been done on the role of DCs in the immune response to *C. parvum*. Studies indicated that DCs contribute to an effector pathway for *C. parvum* clearance, but not through T-cell activation[109]. Infected murine enterocytes and bone-marrow-derived DCs have been shown to express IFN, suggesting that DCs may contribute to the host immune response against *C. parvum*, probably through innate immune mechanisms. Additional work is required to fully understand the role of DCs in activation of T-cells through antigen presentation[109],[110].

Most of the studies of innate immunity against cryptosporidiosis are based on animal models, particularly mouse models. There have been very few studies on immune responses in humans. The few human studies have focused on systemic antibody responses, with a few addressing cell-mediated responses. Other than fecal antibody responses, there have been no studies on the mucosal immune responses in humans. The reader is referred to the recent review by Borad and Ward for a summary of human studies on the innate immune responses to *Cryptosporidium*[111]. Briefly, serum mannose binding lectin deficiency and mbl2 structural gene mutations increased the risk for cryptosporidiosis. In human volunteer studies, TGF-β, TNF-α, IL-1β and IL-4 were expressed in jejunal biopsies but were not associated with symptoms, while IL-15 was expressed in volunteers who did not express IFN-γ and was associated with symptoms. Levels of IL-8, TNF-α, IL-13 and IL-4 but not IFN-γ were elevated in stool samples of children with cryptosporidiosis. Higher levels of CXCL-10 were detected in jejunal biopsies of AIDS patients with cryptosporidiosis compared with controls.

### Adaptive immunity

The adaptive immune system refers to antigen-specific defense mechanisms involving T helper type 1 (Th1) and T helper type 2 (Th2) cells, antibodies and cytokines. Responding to environmental factors produced by antigen-presenting cells, naïve CD4^+^ T cells proliferate and differentiate into effector cells in an antigen-specific fashion when they encounter their cognate antigen. T-cells have been classically described as Th1 cells and Th2 cells, and more recently, Th17 cells and regulatory T-cells (Treg). Currently, there are no data supporting critical roles for Treg and Th17 in *C. parvum* immunity[98]. Th1 CD4^+^ cells confer immunity to infection by intracellular pathogens through production of effector cytokines IFN-γ, Il-12, TNF, and IL-2, while Th2 CD4^+^ cells promote the clearance of multicellular helminths and ectoparasites by producing IL-4, IL-5 and IL-10.

#### Cell-mediated immunity and T-cell responses

Recent studies of cell-mediated immune responses to *Cryptosporidium* in humans are also summarized in the review by Borad and Ward[111]. As expected by the severity of cryptosporidiosis in AIDS patients with low CD4^+^ counts^[e.g.54]^, recent mouse and human studies have confirmed that cell-mediated immune responses play a crucial role in protection against cryptosporidiosis. MHC-II deficient mice are more susceptible to *C. parvum* infection than MHC-I deficient mice. Thus, CD4^+^ T-cells appear to play a dominant protective function in cryptosporidium infection. Human studies have consistently shown that patients with CD4^+^ counts less than 50 cells/mm^3^ are more likely to have a fulminant form of the disease, while those with CD4^+^ counts of >180 cells/mm^3^ have less severe, self-limited disease. Both animal and human studies confirmed that CD4^+^ Th1 response against cryptosporidiosis is mediated mainly by IFN-γ and is MHC II-dependent. As yet, there has been no study showing a direct cytotoxic activity of CD8^+^ T-cells. Unlike the Th1 response, the role of a Th2 response is less clear. In a mouse model, treatment with anti-IL-4 and anti-IL-5 antibodies resulted in an increased level of infection compared to isotype antibody control animals. In addition, neonatal BALB/c mice treated with anti-IL-4 or BALB/c IL-4 gene knockout mice were more susceptible to infection than the appropriate control mice. It may therefore be that there is both a Th1 and a Th2 immune response, with the Th1 response occurring first and a balanced Th1 and Th2 response acting to effectively control the *C. parvum* infection[112]–[117].

#### B-cells and antibody responses

The protective role of antibodies is doubtful because AIDS patients with chronic cryptosporidiosis have high titers of parasite-specific IgG/IgA and mucosal IgA. In addition, studies using B-cell-deficient mice indicate that B-cells are not essential for either resistance to *C. parvum* infection or recovery from infection[98],[111],[118].

#### Cytokines

Although IFN-γ has been shown to be important in both the innate and adaptive immune responses to *C. parvum*, the mechanisms of resistance mediated by this cytokine alone are not completely understood. There are two possible mechanisms: 1) IFN-γ directly inhibits the development of *C. parvum* in cultured enterocytes, where depletion of intracellular iron and inhibition of parasite invasion have been identified as possible mechanisms of action; 2) TNF-α expression can be activated via upregulation of its transcription factor NF-κB by IFN-γ. IL-12 is also an important Th1 cytokine that induces and regulates the production of IFN-γ and also limits *C. parvum* infection *in vivo*. IL-12 knock out mice are susceptible to infection compared to wildtype mice. IL-18 is upregulated in response to *C. parvum* infection, and exogenous IL-18 significantly decreased parasite load while IL-18 knockout mice are susceptible to infection. Additionally, IL-18 plays a regulatory role in the Th1/Th2 balance during *C. parvum* infection[103],[119]–[121].

#### Vaccine/Immunotherapy

*C. parvum* is potentially life-threatening in immunocompromised humans and is a common cause of outbreaks of diarrhea in newborn livestock. To date, no specific or completely effective therapy for cryptosporidiosis has been developed. A vaccine/immunotherapy strategy may be the most reliable and cost effective method with the greatest impact in controlling *C. parvum* infections. Considerable efforts have gone into the analysis of a number of surface glycoproteins thought to be involved in invasion and infection of host epithelial cells, such as *C. parvum* oocyst surface protein 15/60, TRAP-C1, CSL, and *C. parvum* sporozoite surface proteins[122]–[125]. Additionally, the possibility of adoptive immunity has been studied. Recent reports indicated that, when mice received IELs and CD4^+^ T cells from *C. parvum*-infected mice, there was a significant reduction in the parasite load[126]. Furthermore, DNA vaccines that express surface proteins of *C. parvum* have been reported to induce specific cellular and antibody responses to *C. parvum* in mice[127],[128].

## DIAGNOSIS

As the majority of clinical cases of cryptosporidiosis involve the gastrointestinal tract, the most commonly used diagnostic method is the detection of shed oocysts or antigens in stool samples. *Cryptosporidium* is not usually tested for in routine stool parasite antigen and ova tests in China or elsewhere, unless the patient is immunocompromised or there is a known outbreak of diarrhea for which *Cryptosporidium* is suspected. The reader is referred to an overview (Diagnostic Procedures for Stool Specimens. http://www.dpd.cdc.gov) and several reviews on this subject[5],[8],[12],[129]–[131]. When fecal testing is performed, the rate of oocyst shedding and stool consistency may significantly influence the results. Assays may be performed with or without prior fixation, depending upon the method. The more commonly used fixatives are 10% neutral formalin, formalin-ether or sodium acetate-acetic acid formalin. Potassium dichromate may be used as a preservative if oocyst viability or infectivity is to be assessed. Samples may be concentrated to enrich oocysts using flotation such as in a sugar solution with a specific gravity of between 1.15 and 1.20 (e.g. Sheather's solution)[130].

In general, the least costly methods involve bright field staining of fecal samples, such as the Kinyon modified Ziehl-Neelson method and the Auramine phenol method[130], but these methods require interpretation by a trained laboratory technician. Direct and indirect immunofluoresce assays are more costly, but oocysts are readily identified. Both types of these microscopic methods are affected by stool consistency and detection limits are better with a more liquid stool. The Meridian DFA has been used in several studies against which the sensitivity and specificity of other test have been compared^[e.g.131]^. In addition to the indirect and direct immunofluorescence assays, a number of fecal *Cryptosporidium* antigen detection commercial kits are currently available for use with fresh or fixed stool specimens in clinical settings, while molecular methods are more widely used for genotyping and molecular epidemiological studies[46],[130],[132] (see ***Table 2*** for examples of clinical kits and www.k-state.edu/parasitology/reagents for *Cryptosporidium*-related testing reagents). Commercial genotyping kits are now becoming available.

**Table 2:** Examples of methods and commercially available stool assays for *Cryptosporidium* oocysts and antigens

Method	Source/Manufacturers
Bright field stain method	Auramine/phenol stain 2 (hpa-standardmethods.org.uk)
	BBL*TB Kinyoun Stain reagent kit (BD)
	483K (Kinyoun) and 484K Ziehl-Neelson (Medical Chemical Corp.)
	PL-8060 (Pro-Lab Diagnostics)
Immunofluorescent assays	MeriFluor Crypto/Giardia (Meridian Diagnostics, Inc.)
	Aqua-GloG/C kit (Waterborne Inc.)
	DetectIF Cryptosporidium (Shield Diagnostics Ltd.)
	Hydrofluor Combo Giardia/Cryptosporidium (Strategic Diagnostics Inc.)
Enzyme immunoassay (EIA or ELISA)	ProSpec T microplate assay (Alexon-Trend Inc.)
	IDEIA Cryptosporidium (Dako Corp.)
	Color Vue Cryptosporidium (Seradyn Inc)
Immunochromatographic assay	ImmunoCard STAT! Cryptosporidium/Giardia (Meridian Bioscience Inc.)
	Color PAC Giardia/Cryptosporidium (Becton Dickinson)

## TREATMENT

In the immunocompetent individual the signs and symptoms of cryptosporidiosis usually abate in less than 2 weeks. Supportive therapy may be required if diarrhea is excessive[133],[134]. In cases of very severe watery diarrhea, symptomatic treatment, such as liquid and elelctrolyte replacement and agents such as parenteral octreotide may be used to reduce secretory diarrhea in either immuncompetent or imunodeficient individuals[134]. Nutritional support may be required in the event of severe malabsorption. Because cryptosporidiosis is self limiting in immocompetent individuals, it is difficult to determine the true effectiveness of any treatment in this patient population. However, in the USA the antiprotozoal agent nitazoxanide has been approved for use in cryptosporidiosis, but only in immunocompetent children and adult patients[135].

In the immunodefient patient population, a wide array of agents have been used to treat cryptosporidiosis[133],[135] (www.k-state.edu/parasitology/treatment). While some success has been reported in cases using such agents as sinefungin, azithromycin, paromomycin roxithromycin and nitazoxanide, with a reduction in stool frequency, stool volume and parasite shedding being used as measures of success, clinical improvement and complete eradication of the parasite have not been the norm. Even HIV/AIDS patients who appear to have had their *Cryptosporidium* infection cleared following anti-retroviral therapy may relapse if their CD4^+^ counts decline[56], suggesting that the parasite has never been completely cleared. Immune reconstitution appears to be essential for complete resolution of the infection in such patients. The combination of nitazoxanide and highly active anti-retroviral therapy (HAART) appear to be effective, probably because of a direct effect of the antiretroviral aspartyl protease inhibitors on the *Cryptosporidium* life cycle, in addition to the immune reconstitution effect[136].

The variety of parasite stages and the unique location of the pararsitophorous vacuole undoubtedly contribute to the difficulty in rational drug design. In the case of paromomycin, the intracellular parasite stages appear to be accessed from the luminal side of the parasitophorous vacuole but not through the epithelial cell cytoplasm[137]. In addition, there appears to be energy dependent resistance-associated proteins at the host cytoplasm-parasitophorous vacuole interface, probably related to the high expression of CpABC1[138]. Treatment of *Cryptosporidium*-infected cells with paromomycin or cyclosporine A upregulated the expression of *C. parvum* half transporter Cgd1_1350 transcript, raising the possibility that such transporters contribute to intrinsic drug resistance with treatment[139].

## *CRYPTOSPORIDIUM* SPECIES IN CHINA

As in other countries, cryptosporidiosis in China is more commonly seen in infants and young children, is more prevalent in rural than urban areas, shows seasonal peaks[140], and is more frequently seen in patients with immunodeficiency. In addition, a significant number of infected individuals are asymptomatic carriers[141],[142]. In one study using a multiplex immunoassay, it was found that 23% of the pediatric population was seropositive for indices of a recent infection while a similar percentage of the adult population was seropositive for an index of a historic infection[143]. In another study, the incidence of *Cryptosporidium* infection in AIDS patients (4.25%) was lower than generally reported in the West, but as in other countries, cryptosporidiosis was only seen in patients with low CD4^+^ counts (< 100/mm^3^), and the incidence was lower in patients receiving antiretroviral therapy[144].

In China a number of new *Cryptosporidium* subtype families have been found in farm animals and wild animals having the potential for zoonotic transmission[145]–[147]. Furthermore, in a molecular study of *Cryptosporidium* recovered from raw domestic wastewater in Shanghai, 93.7% of the PCR-positive samples had *C. hominis*, 11.1% had *C. meleagridis*, while there were 7 other *Cryptosporidium* species/genotypes. Of 48 subtypes tested, 4 were unique to the area, suggesting that areas of China may have very different populations of *C. hominis* from those reported elsewhere[148]. The clinical significance of these unique subtypes has yet to be determined.
